# Ontology driven integration platform for clinical and translational research

**DOI:** 10.1186/1471-2105-10-S2-S2

**Published:** 2009-02-05

**Authors:** Parsa Mirhaji, Min Zhu, Mattew Vagnoni, Elmer V Bernstam, Jiajie Zhang, Jack W Smith

**Affiliations:** 1 The University of Texas Health Science Center at Houston, Houston, Texas, USA

## Abstract

Semantic Web technologies offer a promising framework for integration of disparate biomedical data. In this paper we present the semantic information integration platform under development at the Center for Clinical and Translational Sciences (CCTS) at the University of Texas Health Science Center at Houston (UTHSC-H) as part of our Clinical and Translational Science Award (CTSA) program. We utilize the Semantic Web technologies not only for integrating, repurposing and classification of multi-source clinical data, but also to construct a distributed environment for information sharing, and collaboration online. Service Oriented Architecture (SOA) is used to modularize and distribute reusable services in a dynamic and distributed environment. Components of the semantic solution and its overall architecture are described.

## Background

**Figure 1:**
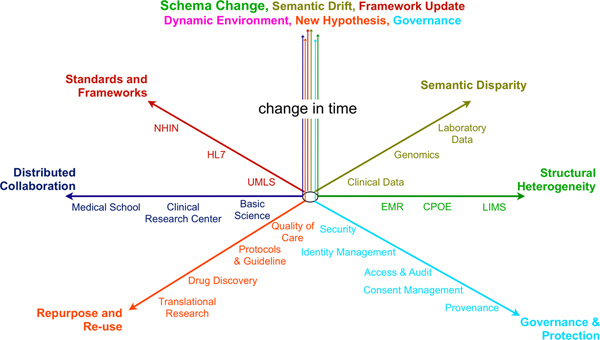
Dimensions of information integration in multidisciplinary and distributed environments such as CTSA.

Understanding, diagnosing, treating, and preventing human disease requires the integration of information and knowledge from all levels of biology including molecules, tissues, organ systems, individuals and populations. Integrating heterogeneous data from multiple sources, and sharing information in a distributed and collaborative environment are challenging informatics problems. Figure 1 illustrates some important aspects of information integration and sharing in multidisciplinary and distributed environments such as CTSA.

Heterogeneous information from semantically and schematically disparate sources must be normalized, integrated and mapped to medical vocabulary systems. Integrated data need to be repurposed to support research and practical applications across different principals, and among different communities, without losing or distorting the semantics and the context of the original data. All these operations need to take place in an environment that is distributed in time and space. They must also comply with policies and regulations relating to the proper conduct of research (consent management, accountability, confidentiality, etc).

Semantic systems and the Semantic Web [1] in particular may be appropriate frameworks for information integration and sharing, and for distributed collaboration on the Internet [2-4]. The Resource Definition Framework [5] (RDF) plus structured ontology development languages such as the Web Ontology Language [6] (OWL) can be used to represent information and knowledge as globally unique resources with formal semantic properties. This enables greater interoperability, integration, repurposing and sharing of information in distributed settings. Information and knowledge represented in RDF and OWL have identical meaning to machines and human experts. These technologies support automatic classification within distributed environments such as the Web.

The Semantic Web and ontologies are frequently cited for their utility in the context of integration of heterogeneous and disparate clinical and biomedical data [7], addressing mainly the structural and semantic heterogeneity axes from the Figure 1. In the rest of this paper we describe our conceptualization of an ontology-driven approach to support not only integration of heterogeneous data but also to enable its sharing among a diverse community of online collaborators. We address multiple dimensions involved in integration, normalization, contextualization, authentication and utilization of shared data.

## Methods

In this section we will describe our approach in terms of ontologies and their application to address information integration challenges in the context of clinical and translational research (*italics *denote references to *concepts defined in the model*).

### 1 – CCTS Environment Model

Typically, research enterprises such as the UTHSC-H can be modeled in terms of their *functional units *(e.g., *Schools*, *Departments*), *affiliates *(e.g., *Collaborating institutions*), *investigators *(as *Individuals*, *Groups*, *Panels*, or *Consortiums*), and *Research Projects *or *clinical activities *around some *principal of science *(e.g., *Rheumatology*, *Genetics*). Each of these entities can be located using their *geospatial *properties and *directory *and *contact information*. Research projects *produce, document, maintain *and *administer *informational and capital resources and variety of *physical or virtual instruments*. Investigators can *participate *in many departments, or research projects, and interact with entities such as *devices, resources *and other investigators.

We developed a generic model of a research enterprise using OWL and instantiated the model with data from the CCTS program at UTHSC-H. The primary purpose of this model is to represent the concepts for a collaborative information-sharing network and to enable governance, communication, information exchange, resource management, audit, and role based security and policy management.

### 2 – Research Documentation Model

Clinical and translational research processes can be modelled as *entities *(e.g., *Patients*), *events *(e.g., *Patient Visit*) that capture and document the status of some *Observations *(e.g., existence of *Signs or Symptoms*). These *events *are frequently associated with some physical forms of *Documentation *(*Survey Form*, *Database*, *Worksheet*, etc).

A generalizable ontology has been developed to enable description of informational resources produced through a *research activity *(e.g., *Surveys*, *Databases*). This model extends the CCTS environment model to track activities and entities within projects and to identify, locate and authenticate access to documented research results

### 3 – Authorization and Control Model

The CCTS Environment model, along with the Research Documentation Model provide the infrastructure for a security, authorization and audit model by describing *investigators*, their *roles *and *membership *in different *research projects *and the *datasets *produced within the project.

The Authorization model extends the Environment and Research Documentation models by asserting access and retrieval rights directly (e.g., *all members of project A can view information from document B of project C*). Alternatively, a reasoner can infer rights using existing facts and axioms (e.g., *user A1 can retrieve all information from form C as he is member of a group that participates in the consortium D, Project C is a project of consortium D, all members of a consortium can access all documents of a given consortium project*).

**Figure 2:**
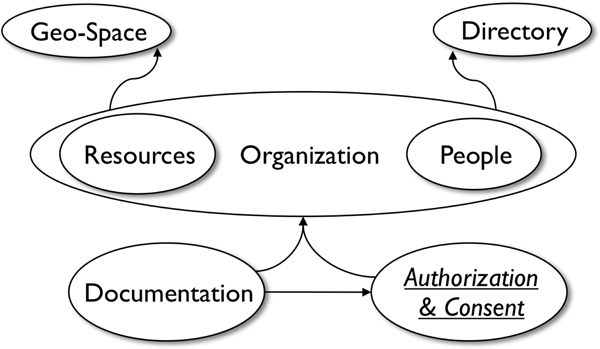
**CCTS Environment Model, Authorization and Consent Model, and Documentation Model and their relationships**. Arrows indicate ontology import (reuse).

A future extension of this model will incorporate concepts required for management of *Patient Consent *and classes of activities that are authorized or disallowed. Figure 2 represents the relationships between the models (ontologies) described so far.

### 4 – Medical Information Model

A semantic application intended to operate in a biomedical environment requires a domain model that describes biomedical concepts (e.g., *Diseases*) and semantic relationships between them (e.g., *All Infectious Disease are Caused by some Infectious Agent*). The Gene Ontology [8] is an example of such a model that provides a controlled vocabulary to describe gene and gene product attributes in any organism.

Although candidate ontologies have started to appear that claim comprehensive and formal description of biomedical concepts (GALEN [9], FMA [10], NCI Thesaurus [11]), these ontologies are too large and complicated to be effectively used and maintained even by trained human experts [12]. Rather than using an extremely large ontology that describes every biomedical concept, we chose to create a smaller, more tractable model that includes relevant and domain-specific concepts extracted from larger ontologies [12]. This model is used to describe and explicate the meaning of information found in our research databases. This model can be replaced, as new more modular ontologies become available, or extended should new types of data be included in the application, and can be aligned with larger ontologies on demand (e.g., GALEN).

### 5 – Integrated Vocabulary Models

Knowledge organization systems (KOS) such as the Unified Medical Language System (UMLS) Metathesaurus are important for consistent documentation and interoperability of health information systems. The following functionality is required for a semantic application to identify correspondence between domain concepts from an ontology (e.g., Fever from the Medical Information Model described above) and a relative concept 'code' from a vocabulary system (e.g., UMLS CUI:C0015967).

▪ A unified method of explicating biomedical vocabularies and taxonomies (i.e., KOS) using formal information representation frameworks such as RDF: Simple Knowledge Organization System [13] (SKOS) is an ongoing W3C standardization effort to support the use of KOS such as thesauri, and taxonomies using the Semantic Web.

▪ A method of representing correspondence between concepts from multiple KOS or between concepts from OWL ontologies and KOS. The method should be able to account for synonymy, hyponymy, and hypernymy and other relationships (such as part-whole relationships, parent-child) frequently found in biomedical KOS.

▪ A method of search and retrieval from a set of existing KOS, to identify relevant concepts, and terms based on a combination of concept names, synonyms, broader/narrower relations, codes, and coding schemes. These methods are traditionally implemented as Vocabulary Services within biomedical applications.

In order to support these features we have developed:

▪ An algorithm to extract medical vocabularies from their source format and to translate to a SKOS ontology. Our current implementation of the method extracts source vocabularies included in the UMLS Knowledge Source. We have also translated all 200 value sets from 15 vocabulary groups pertaining to the Public Health Information Network (PHIN) frameworks to SKOS representation.

**Figure 3:**
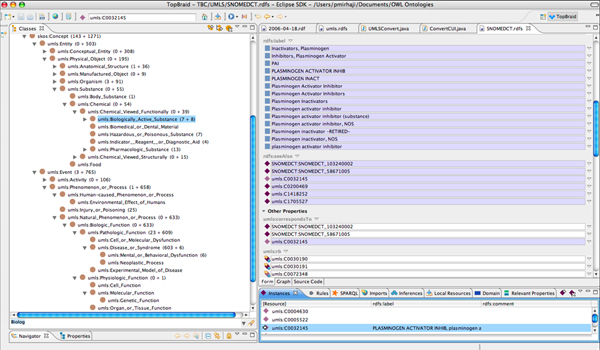
**Snapshot of the SKOS representation of a biomedical concept**. The right side panel represents the SKOS:Concept related to "Plasminogen Activator Inhibitor", synonymous terms associated with it, two distinct SNOMED-CT concepts and a CUI that correspond to it, several other similar or associated concepts (rdfs:seeAlso) from UMLS, and its multiple broader concepts. The Semantic Type (umls:Biologically_Active_Substance) and other relevant classification based on the UMLS Semantic Network are presented in the left panel.

▪ A SKOS model to represent the UMLS Metathesaurus and the UMLS Semantic Network (UMLS-SN) (Figure 3). This also lets us group and classify domain concepts based on UMLS-SN. We have extended the UMLS-SN SKOS model with properties to assert correspondence of OWL concepts or SKOS concepts from different source vocabularies with UMLS Metathesaurus Concept Unique Identifiers (CUI). A reasoner can then infer correspondence between OWL concepts or SKOS concepts from multiple source vocabularies using transitive and functional attributes of the properties (e.g., *if concepts A and B both correspond to C, then A corresponds to B, hence all SKOS:Definition of A also applies to B and vice versa*).

▪ A Web Service [14] with methods to search and navigate the information space in order to identify correspondence of terms, codes, and names used to describe domain concepts, using underlying vocabulary systems.

**Figure 4:**
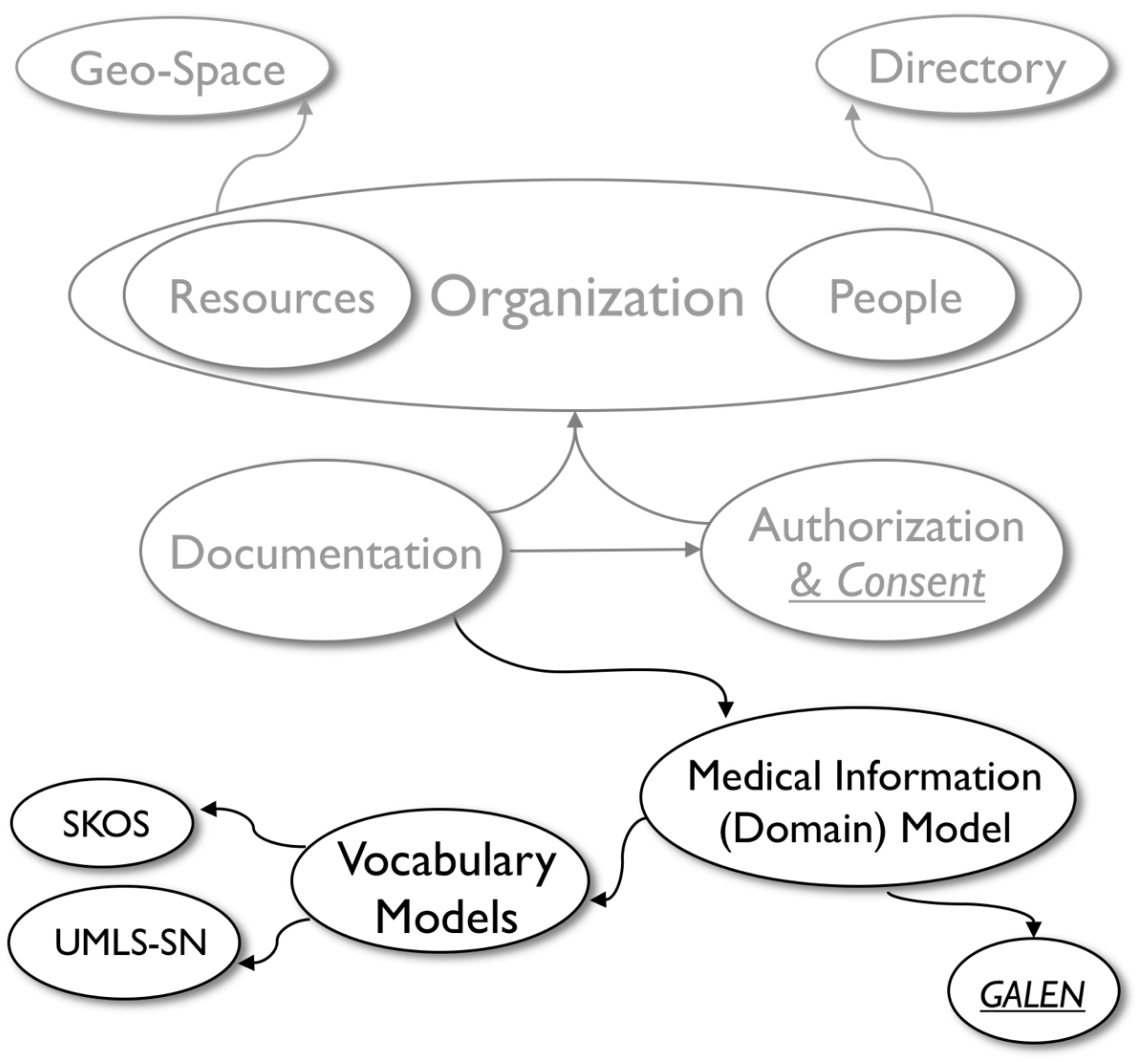
Alignment of Integrated Vocabulary Models and Domain Model with CCTS Environment and Documentation Models.

Once the above mentioned ontologies are aligned (Figure 4) and populated with data from research databases, one can navigate from a high level view of the CCTS, its affiliate departments and research projects to the actual documentation of research and clinical activities concerning specific patients, down to the particular medical observations made for each case and its corresponding vocabulary code. A concept-based navigation strategy enables exploring the integrated information. For example one may start from a concept such as ACE_Inhibitor and navigate to see all information related to administration of any of the ACE inhibitor medications that are known to the system, complications reported and other associated data from an integrated pool of patients with a documented history of ACE inhibitor use, research projects and participating investigators that collected such information, with their departmental and personal contact information, as well as pointers to where the actual research data is stored and located.

The CCTS models described so far were prepared to identify, integrate and make available data that already exists in a research enterprise, but do not explain how new data can be collected and integrated through manual entry or automated data feeds. Next sections of the paper will introduce the models and services that enable provisioning and integrating new datasets.

### 6 – Ontology Driven Survey Design Model (Figure 5)

**Figure 5:**
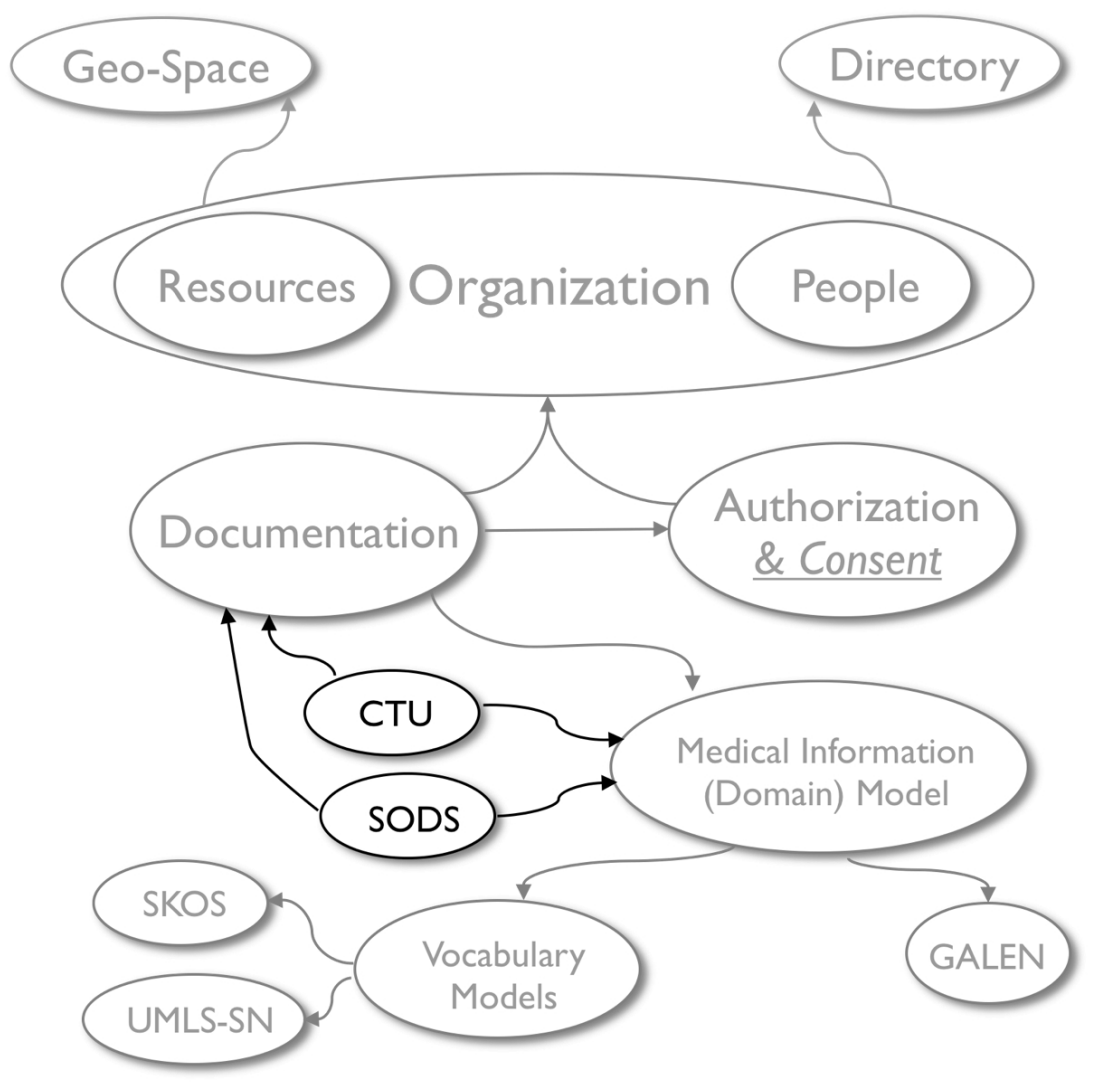
Clinical Text Understanding and SODS Models and overall orchestration of models.

Data entered into a semantic system needs to be mapped to relevant domain ontologies. To automate mapping of new manually-entered data to domain ontologies by users who are not familiar with the underlying technology, we developed a model that describes a typical questionnaire in terms of questions, answer options and some navigation rules to establish relationships between answers and questions (e.g., *if female, ask if pregnant, if pregnant ask about complications and risk factors*).

We developed a semantic application called the Survey On Demand System (SODS) that uses this model, domain ontologies, and KOS that are available to the system to facilitate construction of an ontology driven structured data entry (SDE) tool for questionnaire and survey design. SODS is an interactive tool to help users design custom questionnaires and automatically map all answer options to domain ontologies. SODS is designed to enforce and facilitate consistent use and reuse of domain vocabulary and for longitudinal integration of survey information. Thus, data captured by multiple independent questionnaires will be automatically integrated (e.g., if the same patient has been studied by several independent projects in multiple occasions). Once questionnaire design is complete, identical SDE forms are automatically deployed on a web site, and to a specialized PDA application that collects and submits new submissions. All submissions are mapped to the existing knowledgebase consistently and automatically, without user involvement and are immediately available for semantic querying and integration.

### 7 – Automated Ontology Learning Model

For semantic integration, all incoming data should be mapped to an ontology. But transforming and maintaining a consistent mapping of all incoming data to an integration ontology (such as the model described in section 4) becomes very difficult, especially when multiple disparate and heterogeneous data sources are involved. To automate the integration process, we have developed an ontology-driven method to learn and produce an ontological representation, a "proto-ontology," of any given XML message based on the system's previous experience with the same or similar data sources, and inputs from human experts. The system can optionally merge and integrate multiple disparate datasets into a single proto-ontology, or create custom proto-ontologies for each dataset – that would be mapped to domain ontologies individually. Optionally, the proto-ontology can be processed further by vocabulary services (as described in section 5) to suggest potential correspondence with vocabulary systems or alignment with the appropriate ontologies. Once a human expert modifies parts of the proto-ontology, the system automatically applies the same changes to new data (e.g., persisting user changes even if the new data disagrees, infers inheritance for new siblings of a class, cascades deprecation of concepts to subclasses, instantiates appropriately if equivalencies are established or concepts are merged by human expert, identifies appropriate properties to assert new facts based on updates made by human expert). This approach eliminates all programming usually required for converting XML messages (or relational databases) to custom RDF/OWL representations. Instead, conversion becomes an interactive process, regardless of dataset size or complexity.

### 8 – Clinical Text Understanding

We developed a patient-centric model to describe the syntactic and semantic representations found in a typical clinical text, as it appears in an electronic health record or a reporting system (Figure 5). The model describes relationships between symbols found in a clinical text and concepts from Medical Information Ontology and UMLS-KS. The model has enabled implementation of a minimal syntactic and semantic algorithm to extract concepts, and their relationships from a clinical text, when their representation in the text match patterns described in the model. The output is a formal and explicit representation of the input clinical text as an instantiation of an OWL ontology (rather than a set of concepts extracted from the text, as in conventional NLP methods). Being a formal and self-descriptive model, the output can be immediately integrated or mapped to higher level ontologies, or an existing knowledgebase for inference and ad-hoc querying.

### System implementation

To maximize interoperability, scalability and component reuse, all methods, functions and processes are implemented, deployed and orchestrated as Web Services in a Service Oriented Architecture [14].

## Discussion

The system described in this paper is conceptualized to use formal information and knowledge representation frameworks (i.e., Semantic Web) to construct an ontology-driven information system. All system resources and functions are defined explicitly and dynamically through a set of formal platform-independent reusable models (Figure 5). The use of ontologies and the Semantic Web as the core representation framework is expected to facilitate implementation of an integration platform with the following capabilities:

• Dynamic adaptation and integration of legacy and future systems with the dynamic environment of the CCTS program. It is desirable that the system dynamically adapts to the changes in the CCTS environment (users, tasks, requirements, etc).

• Interoperability and support of distributed collaboration on the Internet, through robust information sharing and exchange.

• Semantic integration of disparate and heterogeneous data, with support of biomedical and standard vocabularies.

• A security, authorization and policy management method tightly integrated to the CCTS environment.

The current system under development at the UTHSC-H School of Health Information Sciences implements prototypes of all above ontologies and methods, and aims to cover a complete cycle of interactions from definition of the CCTS environment and its participating departments and research projects, to integrating and mapping disparate clinical and non clinical research databases and information systems.

The design of our system, and choice of using an ontology-driven system implementation was informed by the following rationale and conceptualizations:

### A) Policy awareness

CCTS is a complex multidisciplinary, distributed and collaborative environment. Thus, information sharing and access to private patient information are extremely sensitive issues governed by ever-changing regulations, policies, and rules that need to be carefully enforced and regularly audited. Our goal was to enable explication and formal representation of such policies in order to enable use of computational algorithms to enforce them. The first step towards construction of such models was a formal representation of the CCTS environment and entities, resources and actors involved. The CCTS Environment, Documentation, and Authorization models enable the system to dynamically and automatically contextualize availability, access, utilization, and retrieval of all informational resources governed by the CCTS program and its collaborators, through combination of constraints based on role, investigator, research project or research question. Although we have not yet created an instance of a generic policy model such as HIPAA, an IRB approval process, or patient consent, our prototype ontologies have set the stage to either create one in near future, or adopt from other research groups. Furthermore, it is expectable that fine-grained and semantic representation of biomedical information and standard vocabularies would support imposing control policies based on semantics. For example, a patient consent may allow use of all patient information except psychological assessment data, for cardiology research but only if it does not involve stem-cells.

### B) Separation of Medical Information Model (domain knowledge) from Medical Vocabulary Model (terminological knowledge)

A design decision was to loosely couple information models used in the system with standard vocabularies. This is expected to allow greater tolerance to changes in both information model and vocabularies used. As a consequence, 1) vocabularies can be presented to the system as a context-independent service and on demand. This not only reduces computational resources required by the system, but also encapsulates the update and versioning of terminological knowledge behind a transparent process; 2) Domain models do not need to commit to, or even be aware of the semantic relationships defined in medical vocabulary systems. A modeller can always use the vocabulary service to identify and extract useful facts from existing terminology systems such as SNOMED-CT and incorporate them as part of a domain model, without having to commit to all or unwanted assertions found in biomedical vocabularies; 3) the same domain model can map to more than one vocabulary at a time, without resulting in inconsistent or unpredictable inferences. This is an important feature not only for interoperability with legacy systems using older versions of a coding scheme, but also for satisfying information exchange needs with systems requiring different sets of terminologies (e.g., SNOMED-CT vs. LOINC for clinical lab test).

### C) Integration of non-structured and structured information into a single unified query interface

A design perspective was to enable querying all information available to the system using a single conceptual framework regardless of the underlying structure of integrated data. For example, a query to identify patient allergy, should be able to find and retrieve relevant information from patient triage data, all nurse or Dr. notes, structured sections of a patient chart or health record, and from all encounters pertaining to that patient. The Clinical Text Understanding model and Automated Ontology Learning models enable transformation and unified representation of all data, whether it be structured or not-structured.

### D) Integration of automated and manual data collection methods

It was important to collect new information through SDE forms that were consistent with the integrated representation used by the system. The SODS model and system were designed with this feature in mind, so that the domain models and relevant vocabularies could be used to facilitate design and deployment of survey forms that can be used for automatic transformation and integration.

The current prototype collects and integrates structured and unstructured data from triage and emergency room chart systems from 8 hospitals in metropolitan Houston area in real time. Furthermore, environmental safety data from 18 sensors and detectors throughout the region, sampling 120 chemicals, particles and meteorological parameters are collected and integrated to the same system in an hourly basis. The integrated data from these sources are primarily used for a regional biosurveillance project. Another transformer service converts and maps patient data from 72 data tables of a multi-center clinical trial on demand. Our current development focus is to design and implement interfaces for model-driven navigation, visualization and user interactions.

## Future work

As the project is in the early stages, there are many unresolved issues to be addressed. We are formalizing an objective and empirical evaluation plan to define, assess and report utility and validity of the methods and technologies developed and used throughout this project.

Performance and scalability issues related to large networks requires optimization as well as maturation of the underlying database and reasoning tools. Partnership with major technology vendors such as commercial database companies has helped break through major technical barriers.

We are also extending our design with an analytical processing technique that enables traversing of large RDF graphs using an ontological representation of dimensions, dimension hierarchies and measures in order to construct multidimensional databases (similar to those of OLAP cubes) asynchronously. This will enable traditional business intelligence and statistical packages to seamlessly interact with the integrated data for mining and analysis.

## Competing interests

The authors declare that they have no competing interests.

## Authors' contributions

PM, JZ, MZ participated in conceptualization, feasibility studies, requirements analysis and design of the architecture. EVB and JWS participated in study coordination, and provisioning the infrastructural requirements of the study. PM designed the overall architecture and methods described in sections 1–4. PM and MZ participated in design and implementation of SODS and UMLS-SKOS components. PM and MMV participated in design and implementation of the automated ontology learning algorithm. PM and EVB drafted the manuscript. All authors read and approved the final manuscript.
